# Framework for estimating renal function using magnetic resonance imaging

**DOI:** 10.1117/1.JMI.9.2.024501

**Published:** 2022-03-15

**Authors:** Masahiro Ishikawa, Tsutomu Inoue, Eito Kozawa, Hirokazu Okada, Naoki Kobayashi

**Affiliations:** Saitama Medical University, Saitama, Japan

**Keywords:** quantitative estimated glomerular filtration rate, magnetic resonance imaging, kidney

## Abstract

**Purpose:**

Nephrologists have empirically predicted renal function from renal morphology. In diagnosing a case of renal dysfunction of unknown course, acute kidney injury and chronic kidney disease are diagnosed from blood tests and an imaging study including magnetic resonance imaging (MRI), and an examination/treatment policy is determined. A framework for the estimation of renal function from water images obtained using the Dixon method is proposed to provide information that helps clinicians reach a diagnosis by accurately estimating renal function on the basis of renal MRI.

**Approach:**

The proposed framework consists of four steps. First, the kidney area is extracted by MRI using the Dixon method with a U-net by deep learning. Second, the extracted renal region is registered with the target mask. Third, the kidney features are calculated based on the target mask classification information created by a specialist. Fourth, the estimated glomerular filtration rate (eGFR) representing the renal function is estimated using a regression support vector machine from the calculated features.

**Results:**

For the accuracy evaluation, we conducted an experiment to estimate the eGFR when MRI was performed and the eGFR slope, which is the annual rate of decline in eGFR. When the accuracy was evaluated for 165 subjects, the eGFR was estimated to have a root mean square error (RMSE) of 11.99 and a correlation coefficient of 0.83. Moreover, the eGFR slope was estimated to have an RMSE of 4.8 and a correlation coefficient of 0.5.

**Conclusions:**

Therefore, the proposed method shows the possibility of estimating the prognosis of renal function based on water images obtained by the Dixon method.

## 1 Introduction

Chronic kidney disease (CKD) is defined as a persistent decrease in estimated glomerular filtration rate (eGFR) to $<60\;\mathrm{mL}/\min\;\mathrm{per}\;1.73\;m^{2}$, the presence of abnormalities suggestive of renal injuries, such as proteinuria, or both.^1^^,^^2^ When CKD progresses, renal replacement therapy is required. Moreover, CKD is also important as an underlying condition related to arteriosclerosis and immunodeficiency, so CKD can be seen to be related to a number of major causes of death.^1^^,^^2^ CKD is a global medical problem affecting from 8% to 16% of the population worldwide.^3^ Due to the wide variety of causes of CKD, there is no specific therapeutic intervention, and it is necessary to detect the disease early and control the risk factors for kidney damage.^3^

When a nephrologist treats a patient with renal dysfunction with an unknown clinical course, the nephrologist often refers to imaging findings for the kidney in addition to laboratory data and medical history. To this end, renal ultrasonography, abdominopelvic x-ray computed tomography, and renal magnetic resonance imaging (MRI) are useful. The nephrologist predicts the potential renal function suggested by the morphology and reflects this potential in the treatment planning.

MRI has a particularly good resolution in terms of soft-tissue contrast and, with the use of appropriate imaging methods, it is possible to obtain detailed information on the internal structure of the kidney, such as the corticomedullary border.^4^ Moreover, MRI can also provide data on the physiological aspects of the kidney. The T2* value of the blood oxygenation level-dependent (BOLD) method is an index of ischemia/hypoxia, which may lead to the progression of CKD, and is significantly correlated with the rate of deterioration of CKD.^5^^,^^6^ The apparent diffusion coefficient value of the diffusion-weighted image is an index related to renal fibrosis and is significantly correlated with the pathological findings of renal biopsy.^7^ As described above, there are high expectations regarding MRI as a noninvasive and multifaceted kidney evaluation method, but MRI has one drawback in that there is no method available for the comprehensive quantification of images.

Traditionally, medical images have been measured by the region of interest (ROI) method. In the ROI method, a rectangular or circular area is selected, and the average value of the signal intensities in the area is used as a representative value. Problems with this method include the possibility that arbitrary decisions made by the observer may involve difficulty in including location information and the fact that only a part of the image can be measured. Pruijm et al.^5^ proposed the 12-layer concentric objects (TLCO) method for analyzing the renal region by dividing this region into 12 layers. The TLCO method is a method of designating the inside and outside of the renal region and analyzing the entire area based on the 12 layers. The external (cortex) and internal (medullary) regions of the kidney have different structures and functions, and the TLCO method considers the peculiar structure of the kidney. In addition, it has been reported that the TLCO method is more stable than the ROI method because the entire renal region is stratified and analyzed simply by specifying the external and internal regions.^8^ However, images that are obtained clinically may be atrophied or deformed when the kidney is damaged and may show individual differences or contain cysts. As such, it is not always possible to divide kidneys evenly into multiple layers, resulting in unstable findings. Therefore, there is a desire for a fully automatic and stable method of kidney analysis.

As a comprehensive quantification method for renal images, Kuo et al.^9^ proposed a method for estimating the eGFR at the time of examination using deep learning and ultrasonic images. In contrast, computer-aided diagnosis (CAD) studies on renal MRI and studies on transplanted kidneys have been reported. Khalifa et al.^10^ proposed a framework that estimates the rejection of transplanted kidneys using the time-series dynamic contrast-enhanced-magnetic resonance imaging (DCE-MRI) method. In this method, images obtained by time-series DCE-MRI are aligned, and the renal region is extracted using the level-set method. The alignment is then corrected, and the cortex is calculated and analyzed based on the brightness from the extracted renal region. Shehata et al.^11^ proposed a method for estimating the rejection of transplanted kidneys using deep learning. The DCE-MRI method requires a contrast medium and cannot be applied in cases of CKD. In addition, it is difficult to extract the cortex based on the brightness in impaired kidneys.

In this study, we attempted to solve the above problems and develop a comprehensive evaluation method for renal MRI that can be applied as a clinical test. By taking advantage of the close relationship between renal morphology and renal function, the water images used in the Dixon method for evaluating the internal structure of the kidney were used to evaluate the target, and the correct label was eGFR, which is an index of renal function. Dixon techniques rely on the difference in resonance frequency between fat and water, and, therefore, fat-only, water-only, in-phase, and out-of-phase images are acquired.^12^ In the kidney, where the water-rich organ parenchyma is surrounded by fat, Dixon or similar fat-suppressed images clearly distinguish the renal parenchyma from the surrounding structure. We propose a method of extracting the renal region from magnetic resonance images with a U-net, converting the extracted renal region into a nonrigid body in the target mask, and then analyzing this region based on the TLCO of the target mask.

## 2 Material and Methods

**Fig. 1:**
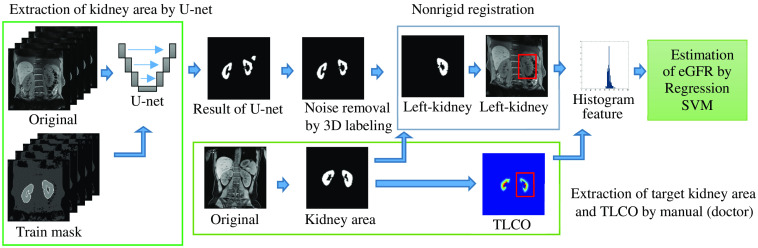
Proposed CAD system for estimation of the glomerular filtration rate slope from MRI using the Dixon method.

The proposed automated framework is shown in Fig. 1. The proposed framework uses the following four steps to process the MRI of the Dixon method:

1. Kidney area segmentation from the surrounding abdominal structures by U-net.
2. Noise removal by three-dimensional (3D) labeling.
3. Nonrigid registration of kidney area and target mask.
4. Calculation of the TLCO.
5. Estimation of the renal function (eGFR) by a regression support vector machine (SVM).

In this paper, the proposed method is the automatic TLCO (A-TLCO) method that compensates for the weaknesses of the previously reported TLCO method and automates the measurement process. The details of the method are described below in order to distinguish the proposed method from the conventional manual TLCO method.

### 2.1 Kidney Area Is Segmented from the Surrounding Abdominal Structures by U-net

**Fig. 2:**
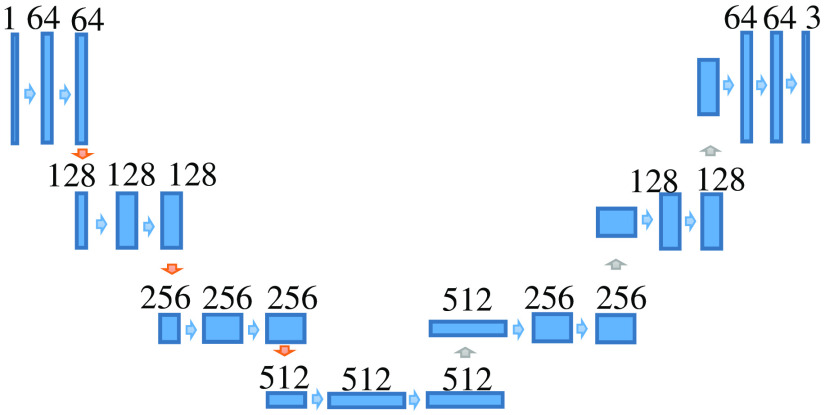
The U-net architecture used for renal region extraction in this study.

**Fig. 3:**
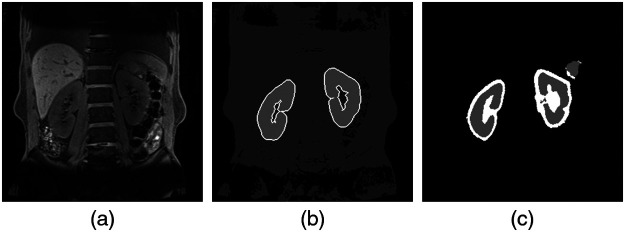
Results of renal region extraction by U-net: (a) original, (b) mask, and (c) result.

Water images of the Dixon method used in this study have a clear renal region. In addition, this section describes a rough extraction of the renal region. Therefore, we have decided to use U-net, which is known to provide good results for the area extraction of medical images.^13^ Coronal sections of Dixon water images were used. There are three to six sliced images per subject, and the image size is 320×320. We used 1201 images of 174 cases. All images were created by a specialist as a renal segmentation image. Figure 2 shows the U-net used for renal region extraction. The network discriminates between three classes: kidney, kidney boundaries, and other tissue. However, there is an imbalanced distribution of samples in the kidney boundaries class as compared with the other tissue class. We used a weighted cross-entropy loss in order to compensate for this imbalance and achieve more accurate learning when training the network. We used softmax with weighted cross-entropy loss for network output and true label comparison. Cost minimization on 50 epochs was performed using the adaptive moment estimation optimizer with a learning rate of 0.0001. The training time for this network was ∼1  h on a workstation with an NVIDIA TITAN RTX GPU × 2. A total of 1201 images were divided into 600 and 601 images, and classification was performed to extract the renal region. The renal function estimation experiment was then performed for all subjects. Figure 3 shows the extraction results obtained by U-net. Figure 3(a) is an input image, and Fig. 3(b) is a teacher image. Figure 3(c) shows the extraction result. It can be confirmed that there are few false positives and that the region close to the renal region can be extracted.

### 2.2 False-Detection Removal by 3D Labeling

**Fig. 4:**
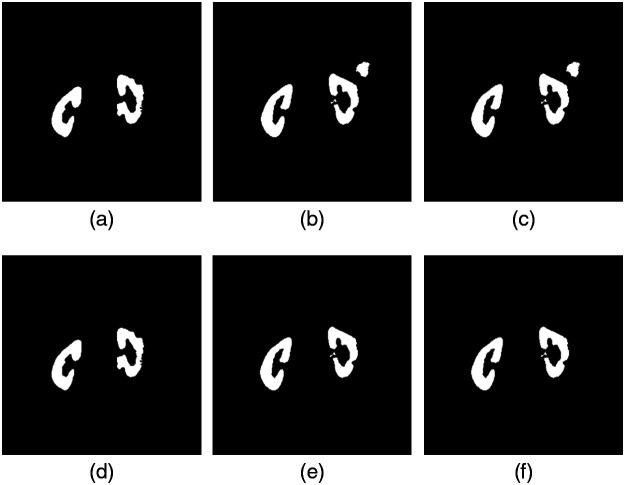
Kidney area obtained by U-net: (a) slice 1, (b) slice 2, and (c) slice 3. Result of the elimination of noise area by 3D labeling: (d) slice 1, (e) slice 2, and (f) slice 3.

Although the renal region detected by U-net was highly accurate, false detection occurred. False positives are extracted for organs other than kidneys, as shown in Fig. 4(b). This may be because when U-net is trained with 64×64 patches, spatial information larger than the patch size is lost. To improve this problem, methods including detecting the location of the kidney by object detection, such as Faster R-CNN^14^ or YOLOv3,^15^ and applying semantic segmentation in a narrow region are considered. However, in this study, the boundary of the kidney by U-net is well discriminated, and there are few false positives for other organs, so there is little need to complicate the process. Therefore, we decided to perform 3D labeling for each subject and exclude areas other than those with a large area. The processed results are shown in Fig. 4. Figures 4(a)–4(c) show the extraction results by U-net. It can be confirmed that false positives occur in areas other than the renal region. Figures 4(d)–4(f) show the results of 3D labeling and exclusion of areas with a 3D area of 2500 pixels or less. This procedure was applied to all 1201 images, and it was confirmed that there were no cases of the renal region being accidentally excluded.

### 2.3 Nonrigid Registration of Kidney Area and Target Mask

**Fig. 5:**
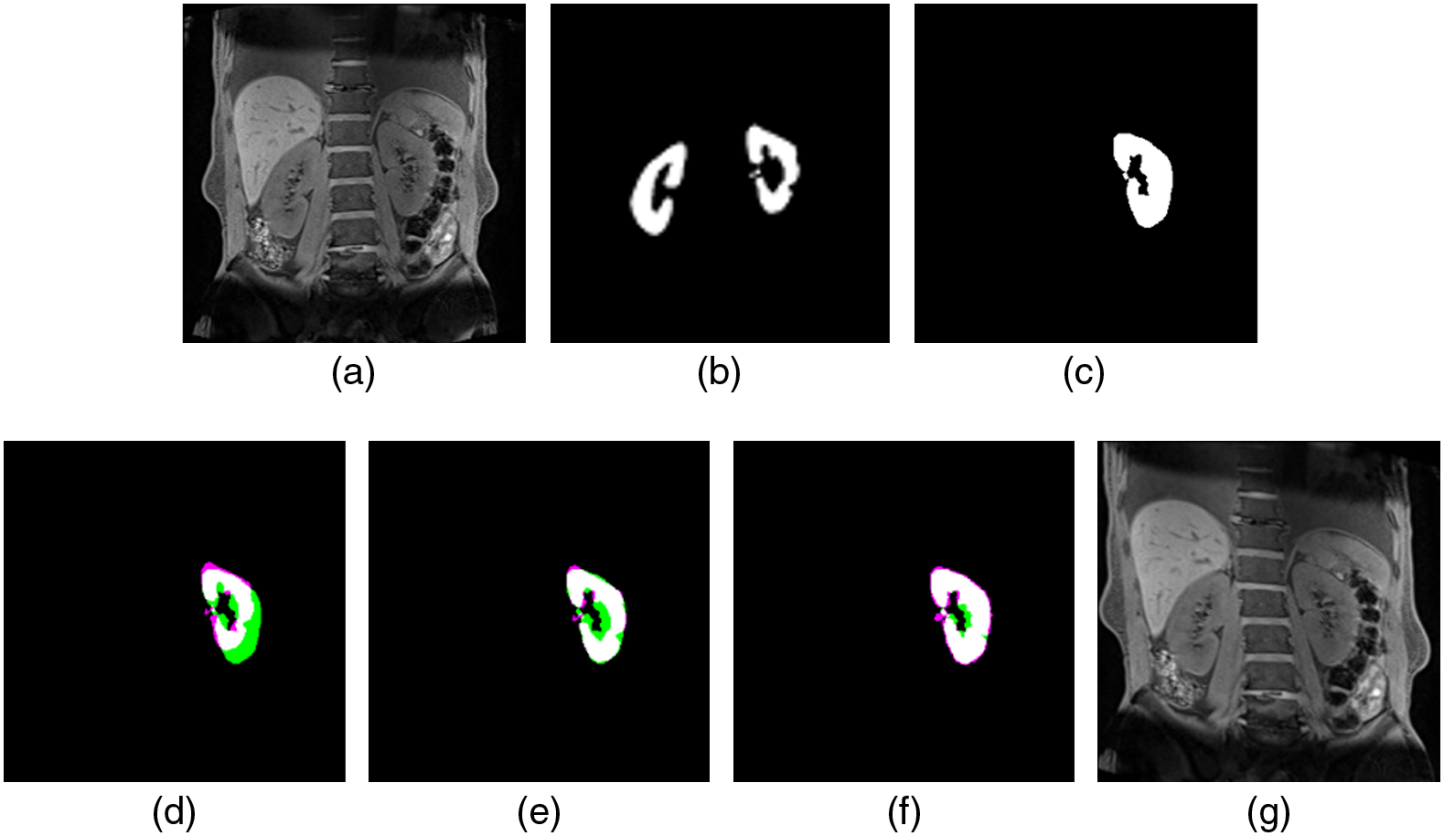
Result of rigid transform: (a) original image, (b) result by U-net, (c) target mask, (d) initial position, (e) affine transform, (f) displacement field, and (g) result of registration.

An effective method for estimating renal function is to analyze the structure of the kidney while considering the kidney anatomically.^5^^,^^16^ However, time and effort are required for a specialist to manually extract the renal region. In addition, manual measurements will result in different results depending on the specialist. Therefore, we propose a method by which to automatically identify the positions of the cortex and medulla of all images by registration of all of the renal regions extracted by U-net to one target mask. The registration method is performed in two steps using a MATLAB function. The first step is a brightness-based affine transformation. The brightness-based registration method calculates the similarity between two images, repeats the affine transformation so that the similarity is high, and estimates the geometric transformation (translation/rotation/scaling/shear) with the highest similarity. The processing results are shown in Fig. 5. Figure 5(a) is the original image. Figure 5(b) shows the results of extracting the renal region by U-net. Figure 5(c) is the target mask. Figure 5(c) is the renal region of a normal renal image extracted by a specialist. A typical patient has two kidneys. In this study, we analyze the kidney with a larger area. In Fig. 5(b), the left kidney is larger. As such, the left kidney is extracted and registered with the target mask. Figure 5(d) shows a pseudocolor image of the target mask and the initial position of the extracted kidney. The pseudocolor image shows the target mask in green, the extracted kidney in magenta, and the overlapping pixels of both in white. The result of the affine transformation is shown in Fig. 5(e). Next, the shape is finely modified by registration using a brightness-based displacement field.^9^^,^^10^ In this paper, the registration is performed using the displacement field based on Thirion’s demons algorithm.^1^^,^^2^ The result of modification of the image shown in Fig. 5(e) using the displacement field is shown in Fig. 5(f). The fine shape has been modified to approach the target mask. Figure 5(g) shows the final registration of the image.

### 2.4 Calculation of TLCO

**Fig. 6:**
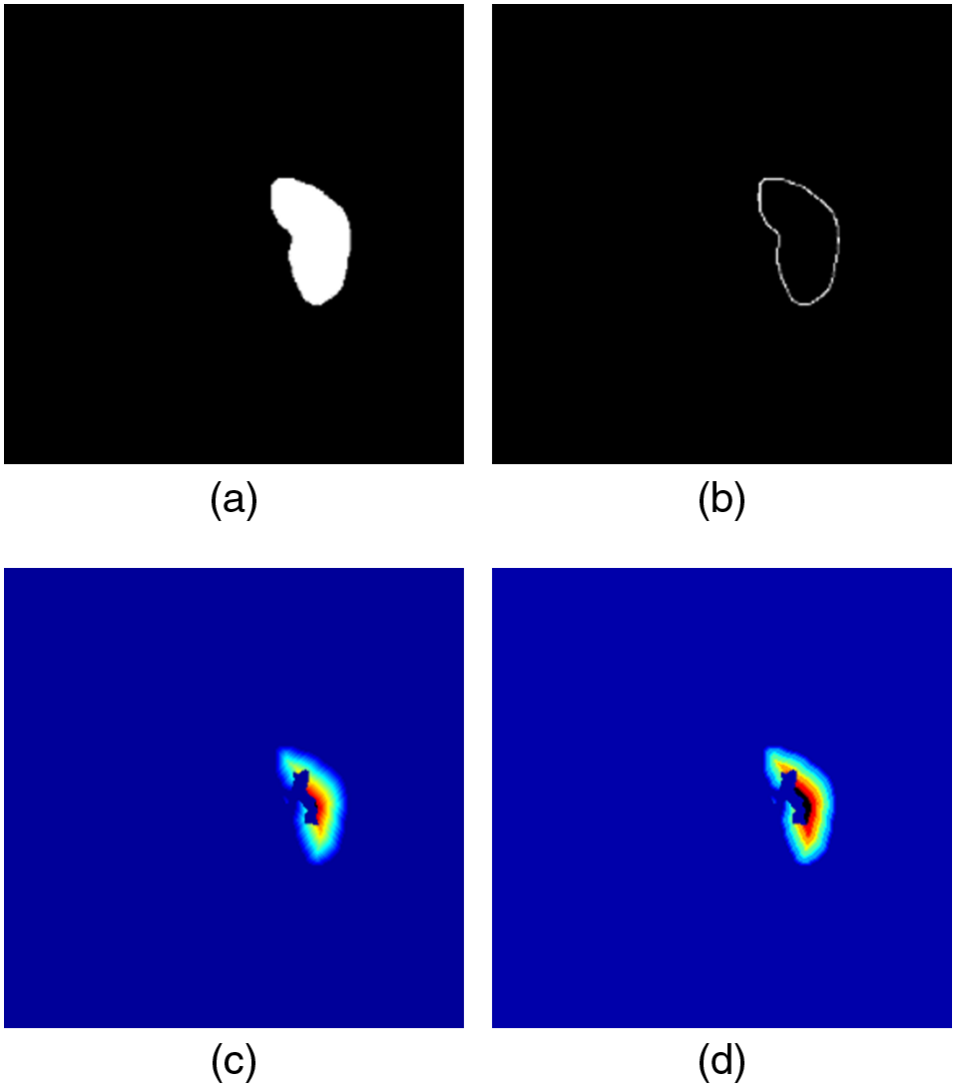
Calculation of the TLCO: (a) mask image, (b) seed image, (c) geodesic distance conversion, and (d) TLCO.

**Fig. 7:**
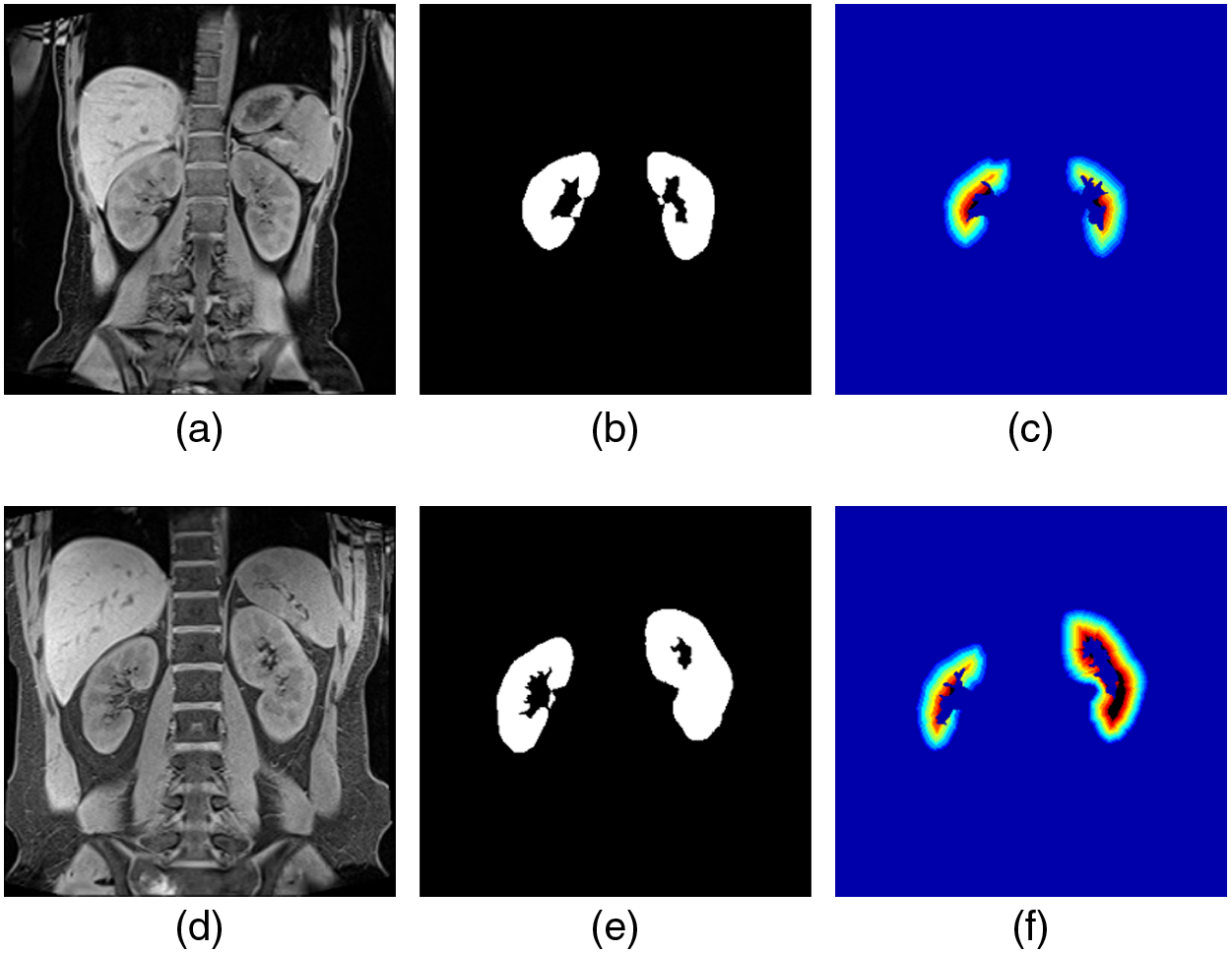
TLCO of the target mask: (a) normal kidney, (b) mask, (c) TLCO, (d) large kidney, (e) mask, and (f) TLCO.

In this study, the TLCO method is used for the analysis of renal function. The TLCO method specifies the inner (medullary side) and outer (cortical side) boundaries of the renal parenchyma and divides the renal region into 12 layers from the outside to the inside. The method for layering is not explained in the TLCO paper.^8^ Therefore, in this study, we calculated the TLCO using geodesic distance conversion.^17^ When geodesic distance conversion inputs a binary image of the renal region and a seed image, a distance conversion image is generated based on the seed image. Figure 6(a) shows a binary image of the renal region extracted by a specialist. Figure 6(b) shows a seed image made by a specialist. Figure 6(c) shows a pseudocolor image of the geodesic distance conversion. However, the image shown in Fig. 6(c) was not obtained by dividing the renal region into 12 layers. Therefore, the result of geodesic distance conversion was normalized into 12 layers. Specifically, the number of layers was divided by the maximum value and then multiplied by 12. The normalized geodesic distance conversion pseudocolor image is shown in Fig. 6(d). The target mask is very important because it directly affects the calculation of the TLCO method. In this study, we decided to use two types of images, a normal kidney image selected by a specialist and the kidney image with the largest region. Since the TLCO method divides the kidney into 12 layers, calculation with a small atrophied kidney is difficult. Therefore, a kidney image with a small area cannot be used as a target mask. Figure 7 shows the results of applying the TLCO method to MRI images of patients. Figure 7(a) is a normal image selected by a specialist. Figure 7(b) shows the results of the specialist extraction of the renal region from Fig. 7(a). Figure 7(c) is a pseudocolor image divided into 12 layers by the TLCO method. Figure 7(d) is an image of the kidney with the largest area. Figure 7(e) shows the result of the specialist extracting the renal region from Fig. 7(d). Figure 7(d) is a pseudocolor image divided into 12 layers by the TLCO method.

### 2.5 eGFR Used to Estimate Renal Function by RSVM

In the TLCO method, we used 12 measurements, one for each layer, to estimate the corticomedullary gradient of MR signal intensity based on the following definitions: cortical (mean of the three superficial layers), medullary [mean of deep layers (layers 8 to 10)], and the gradient [linear fit to the data points (layers four to seven) when plotting MR signal intensity versus % of depth]. It has been reported that the gradient of the MR signal intensity correlates with the eGFR.^8^ As chronic damage progresses in the kidney, the difference between the outer and inner layers in the internal structure disappears. The nephrologist evaluates these changes from the images. Therefore, computing the gradient of MR signal intensity from the outer to inner layers, the fourth to seventh layers provides an index that replaces the human evaluation index. The process has been explained in previous reports, and this index is known to be useful in the evaluation of kidney images.^5^^,^^16^^,^^18^ In this study, we decided to use machine learning to estimate renal function. At this time, eGFR is a continuous value. Therefore, eGFR is estimated using a regression support vector machine (RSVM). In addition, the TLCO method uses the average value of each layer. However, in this study, the histogram of each layer is used as a feature. The number of bins in the histogram is 50. The finally calculated features are the mean, kurtosis, skewness, median, a median of each TLCO (12 feats) and histogram (50 feats) of all renal regions, and the histogram of each TLCO (12×50=600  feats) was calculated. In addition, a noise-removed image was created using a median filter. The noise-removed image was created by applying the median filter once and twice. The feature was calculated from the noise-removed image by the same method. Therefore, the number of features calculated as a feature candidate is 1998.

## 3 Results

### 3.1 Patient Data and MRI Acquisition Protocol

CKD patients with a sufficient number of visits for the treatment and evaluation of CKD (more than three times during a period of at least 1 year) were enrolled (n=165, 105 men and 55 women, aged from 19 to 87 years, and with a mean age of 60±15  years). The proposed framework has been tested on two-dimensional water images of the Dixon method data sets collected from 165 subjects. Magnetic resonance examinations were performed on a 3.0 T (Skyra, Siemens Healthcare, Erlangen, Germany) scanner using a spine coil and an 18-channel phased-array body coil. Coronal 3D T1-weighted volume-interpolated breath-hold examination Dixon images were generated from in-phase, out-of-phase, fat-only, and water-only images.^12^ The MRI parameters were as follows: echo time (TE); delta TE (= 2.46 ms); delta (= 1.23); repetition time (= 5.35 ms); flip angle (= 10 deg); field of view (=360×360×144  mm); recon matrix (= 320); and respiratory compensation (= breath-hold). During the observation period, blood and urine data were sampled every 3 to 6 months. The eGFR was calculated using the formula for Japanese patients,^19^ i.e., eGFR ($\mathrm{mL}/\min/1.73\;m^{2}$) = 194 × Cr − 1.094 × age − 0.287 (× 0.739 for female cases). Since eGFR always decreases, we performed a linear approximation using blood test results from multiple time points and calculated the eGFR at the date of imaging. We also estimated the rate of decline in eGFR using the same linear approximation method and subsequently defined the “annual rate of decline in eGFR = eGFR slope” as an index of CKD prognosis. Urinary protein (UPro) levels were noted and corrected for urinary creatinine (urine protein: creatinine ratio, g:g). The Institutional Review Board of Saitama Medical University approved this research and its publication in accordance with the Declaration of Helsinki and the Japanese guidelines for clinical research (Approval Nos. 12065, 18107, 19048.01, 20107.01).

#### 3.1.1 Kidney segmentation

In this study, U-net is used to extract the renal region. To determine the hyperparameters of U-net, experiments were conducted with patch sizes of 32×32, 64×64, and 128×128 and two to four layers. In the experiment, 600 of the 1201 water images of the Dixon method were used for learning, and the extraction accuracy of 601 images was evaluated. The segmentation accuracy is evaluated using the Dice similarity coefficient, characterizing the agreement between the segmented and ground truth regions. The dynamic susceptibility contrast (DSC) is defined by the following expression:

$$\text{Dice coefficient}=\frac{2\mathrm{TP}}{2\mathrm{TP}+\mathrm{FP}+\mathrm{FN}},$$

where TP, FP, and FN denote the true positive, false positive, and false negative segmentation results, respectively. As illustrated in Table 1, the closer the DSC is to 1, the better the segmentation. To obtain the ground truth in our experiments, a medical specialist delineated the kidney borders.

**Table 1:** Confusion matrix.

		Ground truth	
		Kidney area	Other area
U-net result	Kidney area	TP	FP
	Other areas	FN	TN

In addition, Table 2 shows the accuracy of the two-class (renal region and other tissues) segmentation, and Table 3 shows the accuracy of the three-class (renal region, renal region boundary, and other tissues) segmentation. In addition, erosion and dilation treatment was added as needed. The best accuracy was 87%. The most accurate results were for the three-class mask (renal region, renal region boundary, and other tissues), three layers of U-net, and a patch size of 64×64. Similar results were obtained for four layers of U-net and a patch size of 128×128. In this study, we decided to use three layers of 64×64 in consideration of learning time.

**Table 2:** Accuracy of two-class segmentation result by U-net.

Two-class		The number of layers of U-net		
		2 (%)	3 (%)	4 (%)
Patch size	32 × 32	77	79	80
	64 × 64	79	82	82
	128 × 128	77	82	83

**Table 3:** Accuracy of three-class segmentation result by U-net.

Three-class		The number of layers of U-net		
		2 (%)	3 (%)	4 (%)
Patch size	32 × 32	82	84	85
	64 × 64	84	87	86
	128 × 128	82	86	87

#### 3.1.2 Comparison of the TLCO and A-TLCO methods

In this study, we estimate the eGFR by multiple regression analysis using the average value of each layer of the TLCO calculated from water images of the Dixon method for comparison with the A-TLCO method. The estimated results by the TLCO method are shown in Table 4. Multiple water images of the Dixon method were obtained for each subject. In this study, we analyze the kidney with the largest area for each subject. UPro is known to correlate with renal function in urine and blood tests. The correlation coefficient is shown in Table 4. Upro had a negative correlation of −0.32. On the other hand, the result of manually creating TLCO and estimation by multiple regression analysis was 0.49. The TLCO method results correlate better with the eGFR as compared with the UPro. On the other hand, the correlation coefficient estimated using multiple regression analysis calculated by the A-TLCO was 0.46. Therefore, it was clarified that the proposed method enables TLCO analysis without the need for a specialist to select the renal region.

**Table 4:** Correlation of eGFR by multiple regression analysis.

	UPro	Manual	Affine	Affine&Int
Accuracy of registration	—	—	0.9	0.88
Correlation	−0.32	0.49	0.46	0.42

#### 3.1.3 Estimation of renal function by RSVM

The accuracy of the A-TLCO method was evaluated using an RSVM and a regression random forest (R-RF). The root mean square error (RMSE) and correlation coefficient were used for evaluation. The RMSE was calculated as the accuracy evaluation of continuous values, as follows:

$$\mathrm{RMSE}=\sqrt{\frac{1}{N}\overset{N}{\underset{i=1}{\sum }}{\left(F_{i}-T_{i}\right)}^{2}},$$

where N is the number of estimated values, F is the estimated value, and T is the true value. In addition, we decided to calculate the correlation coefficient to verify the clinical significance of the estimation

$$\text{Correlation}(F,T)=\frac{1}{N-1}\overset{N}{\underset{i=1}{\sum }}(\frac{F_{i}-\mu_{F}}{\sigma_{F}})(\frac{T_{i}-\mu_{T}}{\sigma_{T}}),$$

where μ is the average and σ is the standard deviation. A-TLCO registration was performed with a target mask. In this case, the accuracy may differ depending on the target mask. The A-TLCO divides the renal region into 12 layers. Since dividing the kidney into 12 layers is difficult if the kidney is atrophied, we decided to use two types of kidneys: kidneys in good condition that are closest to normal and kidneys that have the largest area among the normal cases as selected by a specialist. In addition, for the registration method, we compared the affine transformation and the result of applying the displacement field-based registration method in addition to the affine transformation. Feature selection was performed using the forward step-wise method. Subject-based cross-validation was used for accuracy evaluation. Table 5 shows the results of eGFR at the time of MRI imaging. The maximum value of the eGFR used in this study is 113, and the minimum value of the eGFR is 9.4. The best estimation correlation coefficient of eGFR was obtained by using a kidney with a large area as the target mask, applying affine and displacement field-based transformations for registration, and using RSVM, with an RMSE of 11.99 and a correlation coefficient of 0.83. An expert manually calculated 165 TLCO and estimated these values using RSVM results with an RMSE of 15.07 and a correlation coefficient of 0.69. The RSVM models were created using MATLAB 2019a in the experiment. The training option and parameters used for training were as follows: kernel = rbf, box constraint = iqr(Y)/1.349, and ε = iqr(Y)/1.349. Here, iqr(Y) is the interquartile range of response variable Y. The kernel width parameter σ was determined by a heuristic subsampling procedure. We compared the SVM with the R-RF. The number of trees was set to 300, and the parameters were used by default. The results obtained using the R-RF indicated that the RMSE of the eGFR was 14.48 and that the correlation coefficient was 0.76. In addition, we compared the results using the deep learning of GoogLeNet. To use GoogLeNet in this study, the last fully connected layer, softmax, and the classification output of the network were deleted, and the fully connected layer and regression layer of output 1 were added. GoogLeNet uses the result of learning with ImageNet^20^ as transfer learning. The training option and parameters used for training were as follows: initial learning rate = 0.005, Max Epochs = 100, l2reg = 0.0001, and Mini Batch Size = 64. Subject-based cross-validation was used for accuracy evaluation. The results obtained using GoogLeNet indicated that the RMSE of the eGFR was 21 and the correlation coefficient was 0.23. We also compared the method with deep learning and SVM (GoogLeNet + SVM), which has recently been the focus of attention. With the pretrained GoogLeNet, the high-dimensional features that occur after obtaining the deep features of the MRI images are reduced with the neighbor component analysis (NCA) method.^21^^,^^22^ The accuracy was evaluated by SVM using the features obtained by NCA. The results obtained using GoogLeNet + SVM indicated that the RMSE of the eGFR was 21 and that the correlation coefficient was −0.01. Table 6 shows the estimation results for eGFR slope, which indicates the prognosis of renal function. The maximum value of eGFR slope used in this study is 50.5, and the minimum value is −27.6. The best estimation correlation coefficient of the eGFR slope was obtained by using a kidney with a large area as the target mask, applying affine and displacement field-based transformations for registration, and using an RSVM with an RMSE of 4.8 and a correlation coefficient of 0.5. A similar correlation coefficient was obtained when using an RSVM with an RMSE of 4.58, and a correlation coefficient of 0.5. An expert manually calculated 165 TLCO and estimated the TLCO using RSVM results with an RMSE of 4.82 and a correlation coefficient of 0.45. GoogLeNet was calculated to obtain an RMSE of 5.35 and a correlation coefficient of −0.09. GoogLeNet + SVM was calculated to obtain an RMSE of 5.25 and a correlation coefficient of −0.11.

**Table 5:** Accuracy of eGFR by machine learning.

Discriminator	Mask type	Registration method	eGFR (present)		The number of features
			RMSE	Corr	
Regression-SVM	Normal kidney	Affine	**12.17**	**0.83**	**21**
		Affine & Disp	14.06	0.74	13
	Large kidney	Affine	12.89	0.8	19
		Affine & Disp	**11.99**	**0.83**	**31**
R-RF	Normal kidney	Affine	14.96	0.71	7
		Affine & Disp	15.47	0.67	8
	Large kidney	Affine	14.45	0.76	8
		Affine & Disp	14.48	0.76	8
Regression-SVM	Manual extraction		15.07	0.69	11
GoogLeNet			21	0.23	—
GoogLeNet + SVM			21	−0.01	—

Note: Bold value is the discriminator with the highest correlation coefficient.

**Table 6:** Estimation accuracy of eGFR slope by machine learning.

Discriminator	Mask type	Registration method	eGFR slope		The number of features
			RMSE	Corr	
Regression-SVM	Normal kidney	Affine	4.87	0.4	8
		Affine & Int	4.8	0.48	9
	Large kidney	Affine	4.86	0.42	5
		Affine & Int	**4.8**	**0.5**	**14**
R-RF	Normal kidney	Affine	4.68	0.44	5
		Affine & Int	**4.58**	**0.5**	**8**
	Large kidney	Affine	5.00	0.28	4
		Affine & Int	4.73	0.42	5
Regression-SVM	Manual extraction		4.82	0.45	7
GoogLeNet			5.35	−0.09	—
GoogLeNet + SVM			5.25	0.11	—

Note: Bold value is the discriminator with the highest correlation coefficient.

### 3.2 Selected Features of the Proposed Method

**Fig. 8:**
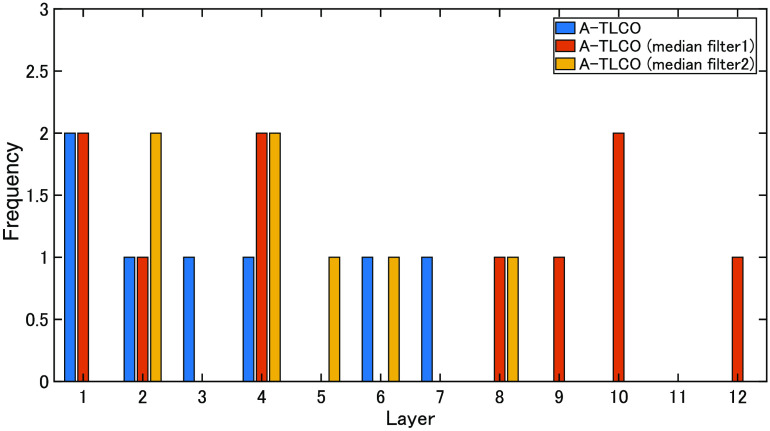
A-TLCO histogram features selected by eGFR estimation.

In this study, 1998 features were calculated, and feature selection was performed using the forward step-wise method. The eGFR estimation required an average of 14 features. The least-cases scenario involved seven features, and the most-cases scenario involved 31 features. Table 5 indicates the number of features required for convergence. Table 7 shows the features selected for estimating eGFR (RSVM + Large kidney + Affine & Int). The histograms calculated from the entire mask were selected from 7 out of 31 features. However, most of the histograms were created for each A-TLCO layer and 24 of the 31 features. Figure 8 shows which layer was selected in the TLCO layer-by-layer histogram. At this time, layer 1 is the outermost layer, and layer 12 is the innermost layer. Layers 4 to 6 are the boundaries between the cortex and the medulla. Although the features are selected from the histogram of each layer, relatively many bins of the layers corresponding to the inside and outside are selected. This may indicate that changes in the cortex and medulla are effective. In addition, many features of the image from which noise was removed by applying a median filter were selected.

**Table 7:** Features selected for eGFR estimation.

Feature name	Number of features
Mean, kurtosis, skewness, median (4 × 3 = 12 feats)	0
Histogram of kidney (50 feats)	1
Histogram of kidney of median filter one time (50 feats)	1
Histogram of kidney of median filter two times (50 feats)	5
Median of each layer of A-TLCO (12 × 3 = 36 feats)	0
Histogram of each layer of A-TLCO (600 feats)	7
Histogram of each layer of A-TLCO of median filter one time (600 feats)	10
Histogram of each layer of A-TLCO of median filter two times (600 feats)	7

**Fig. 9:**
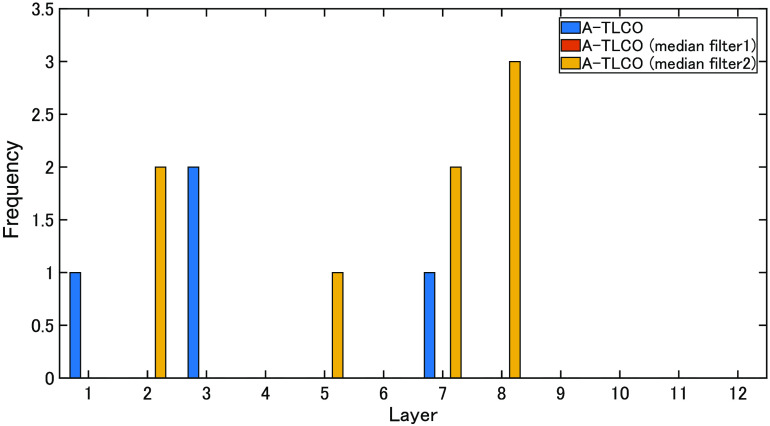
A-TLCO histogram features selected by slope estimation.

Table 7 shows the number of features required for convergence of the eGFR. Table 8 shows the features selected for estimating the eGFR slope (RSVM + Large kidney + Affine & Int). The mean, kurtosis, skewness, median, and histogram calculated from the entire mask were selected from 2 of 14 features. However, most of the histograms were created for each A-TLCO layer and 12 of the 14 features. Figure 9 shows which layer was selected in the TLCO layer-by-layer histogram. As few as 12 features are selected, and relatively many bins of the layers corresponding to the inside and outside are selected. In addition, many features of the image from which noise was removed by applying a median filter were selected.

**Table 8:** Features selected for eGFR slope estimation.

Feature name	Number of features
Mean, kurtosis, skewness, median (4 × 3 = 12 feats)	1
Histogram of kidney (50 feats)	0
Histogram of kidney of median filter one time (50 feats)	1
Histogram of kidney of median filter two times (50 feats)	0
Median of each layer of A-TLCO (12 × 3 = 36 feats)	0
Histogram of each layer of A-TLCO (600 feats)	4
Histogram of each layer of A-TLCO of median filter one time (600 feats)	0
Histogram of each layer of A-TLCO of median filter two times (600 feats)	8

## 4 Discussions

### 4.1 Performance of the Proposed Method

In this study, renal function was estimated using water images of the Dixon method. The eGFR accuracy was 0.38 when estimated by multiple regression analysis using the average value for each layer. On the other hand, when a normal kidney with a large area was used as the renal region and affine transformation and displacement field registration were used for registration, the RMSE was 11.99 and the correlation coefficient was 0.83, which is a large improvement. Large kidneys tended to have better RMSE and correlation coefficients than normal kidneys. For comparison, features were calculated and evaluated using the TLCO manually extracted by a specialist. The TLCO method has an RMSE of 15.07 and a correlation coefficient of 0.69. Better results were obtained with the A-TLCO method than with the TLCO method. It is thought that the reason for this is that the value provided by the TLCO method varies because the renal region is manually divided into layers and dividing small kidneys, such as atrophic kidneys, into 12 layers is difficult. The results obtained by GoogLeNet had an RMSE of 21 and a correlation coefficient of 0.23. Using GoogLeNet + SVM did not provide good results, giving an RMSE of 21 and a correlation coefficient of −0.01. It became clear that estimation by simple deep learning was difficult. This probably indicates the small number of data used for learning as well as the difficult estimation of information that specialists cannot recognize from images, using deep learning. However, using the A-TLCO method, we found that eGFR could be estimated with high accuracy from water images of the Dixon method.

A similar experiment was performed on the eGFR slope. The eGFR slope was calculated using a normal kidney with a large area as a renal region, and affine transformation and displacement field registration were used for registration. The RMSE was 4.8, and the correlation coefficient was 0.5. In the estimation of the eGFR slope, a correlation coefficient of 0.5 was also obtained using R-RF. However, the estimated eGFR slope tended to have a significantly lower correlation coefficient than the eGFR estimation. The TLCO manually extracted by a specialist was also evaluated using the same method. The RMSE was 4.82, and the correlation coefficient was 0.45. Better results were obtained with the A-TLCO method. The results obtained by GoogLeNet had an RMSE of 5.35 and a correlation coefficient of −0.09. Using GoogLeNet+SVM did not provide good results, giving an RMSE of 5.25 and a correlation coefficient of 0.11. It was again clear that estimation by simple deep learning was difficult. However, it was found that the prognosis of renal function can be estimated to some extent using the A-TLCO method, even using water images of the Dixon method. In order to improve the accuracy of eGFR slope estimation, it is necessary to consider different MRI images, such as the T2* map generated by BOLD MRI.

### 4.2 Adequacy for Clinical Application of Selected Features of the Proposed Method

Nephrologists have empirically predicted renal function from renal morphology. In diagnosing a case of renal dysfunction of unknown course, acute kidney injury and chronic kidney disease are diagnosed from blood tests and an imaging study including MRI, and an examination/treatment policy is determined. For example, the discrepancy between the predicted eGFR from the images and the actual eGFR is known to be large in the case of acute kidney injury, while in the case of chronic kidney injury, the discrepancy is small. In addition, MRI is used as a reference for predicting renal function recovery by treating patients with acute kidney injury. However, nephrologists empirically predict renal function based on the size of the kidney, surface irregularities, and the clarity of the boundary between the cortex and medulla, and quantitative evaluation is difficult. On the other hand, this study is expected to allow quantitative assessment and reproducibility to diagnostic imaging by MRI. The proposed method is a framework for quantifying the state of the kidney and can be applied to various types of MRI, such as T2* mapping. Therefore, the proposed method is expected to become a new imaging method for evaluating various physiological and pathological aspects of the kidney.

## 5 Conclusion

We proposed the A-TLCO method to estimate renal function from water images obtained using the Dixon method. The proposed method afforded an RMSE of 11.99 and a correlation coefficient of 0.83 for eGFR estimation, an RMSE of 4.8 and a correlation coefficient of 0.5 for eGFR slope, which is an indicator of CKD prognosis. These results confirmed that the reliability was high and the accuracy was dramatically improved compared with the conventional TLCO method.

The *de facto* standard for MRI measurement is the ROI method. Since the kidneys are concentrically arranged with functional and anatomical distinctions, it has been reported that the TLCO method provides more information than does the ROI method.^8^ In this paper, by adding auto-annotation and registration processing using the mask, as well as devising the features used in the estimation, we have been able to greatly improve the accuracy of the estimation using the features obtained by the conventional TLCO method.

MRI methods that can acquire the status of hypoxia and fibrosis have been reported, just as water imaging can image the internal structure of the kidney. Unlike x-ray computerized tomography and ultrasonography, MRI can also provide the physiological and functional aspects of the kidney along with positional information. As such, the proposed method of comprehensive MRI quantification is expected to afford an important imaging analysis method that can provide useful information to clinicians.
